# FORTCARE-MCI study protocol: evaluation of Fortasyn Connect in the management of mild cognitive impairment in primary care

**DOI:** 10.3389/fneur.2024.1434210

**Published:** 2024-10-03

**Authors:** Enrique Arrieta, Pablo Baz, Guillermo García-Ribas

**Affiliations:** ^1^Segovia Rural Health Center, Neurology Working Group of SERMERGEN, Segovia, Spain; ^2^North Periurban Health Center of Salamanca, Neurology Working Group of SERMERGEN, Salamanca, Spain; ^3^Hospital Universitario Ramón y Cajal, Madrid, Spain

**Keywords:** mild cognitive impairment, primary care, treatment, Fortasyn connect, Alzheimer’s disease

## Abstract

**Background:**

Neuropsychiatric symptoms are prevalent in patients with mild cognitive impairment (MCI) and are predictive of the conversion to dementia. Fortasyn Connect, a medical food, has shown efficacy in managing cognitive and behavioral symptoms associated with MCI. Early diagnosis and intervention in primary care are essential for managing MCI. However, real-world prospective studies assessing Fortasyn Connect in MCI are still limited.

**Methods:**

This observational, multicenter, prospective study will enroll 150 patients recently diagnosed with MCI by primary care physicians across several regions in Spain. Participants will be followed-up over a 12-month period, with assessments at baseline, 6 months, and 12 months, as per clinical practice. The study aims to evaluate the impact of Fortasyn Connect on neuropsychiatric symptoms, cognition, and health-related quality of life (HRQoL) using validated neuropsychological tests and machine learning (ML) techniques. The primary outcome measure will be changes in neuropsychiatric symptoms using the Neuropsychiatric Inventory Questionnaire (NPI-Q) at 6 months. Secondary outcome measures include further changes in the NPI-Q at 12 months, and changes in cognition (Fototest, and clock-drawing test) and HRQoL (EQ-5D-5L) at 6 and 12 months. Exploratory outcomes will assess speech using an artificial intelligence (AI)-enhanced ML tool, with a correlation analysis of these findings with traditional neuropsychological test results.

**Conclusion:**

This study will provide evidence of the effectiveness of Fortasyn Connect in a real-world setting, exploring its potential to stabilize or improve neuropsychiatric symptoms, cognition, and HRQoL in MCI patients. Results will also contribute to the understanding of AI and ML in identifying early biomarkers of cognitive decline, supporting the timely management of MCI.

## 1 Introduction

Mild cognitive impairment (MCI) represents a transitional state between normal aging and dementia. Individuals with MCI have cognitive deficits beyond those expected for age and education but are still able to function independently (1, 2). These impairments encompass memory, attention, language, and executive functions (3). The prevalence of MCI varies among studies, but recent estimates suggest that approximately 15% of community-dwelling adults aged 50 years and older suffer from MCI (4). This clinical syndrome is due to Alzheimer’s disease (AD) in nearly 50% of the cases (5). The annual conversion rate to dementia of patients with MCI falls within the range of 10–33% (6), and in 2–3 years, approximately half of MCI patients transition to dementia (7–9). As the global population ages, the burden of MCI and dementia increases (10, 11), considerably affecting the health-related quality of life (HRQoL) of patients and their families (12–14).

Timely diagnosis and early intervention could result in delaying disease progression and worsening of symptoms, leading to improvements in HRQoL and resource savings (10). However, despite its widespread prevalence, MCI is still underdiagnosed within clinical practice (15, 16). Primary care physicians are on the front line of MCI diagnosis and management (17, 18). Studies have shown that most patients with dementia were initially diagnosed by a clinician other than a dementia specialist (19). While primary care physicians are responsible for the majority of initial MCI and dementia diagnoses, a significant proportion —nearly 40%— expressed discomfort in diagnosing AD or other dementias, as revealed by a 2019 survey conducted by the Alzheimer’s Association (20). This might be due to all the barriers associated with the diagnosis in primary care, including short appointments and lack of resources (21). Primary care physicians might benefit from training to apply validated and easy-to-use tools to help identify the first symptoms and signs of MCI (22).

Neuropsychiatric symptoms, also known as behavioral and psychological symptoms, may precede the onset of cognitive decline (23, 24) and increase with disease severity (25). These symptoms are highly prevalent in patients with MCI (26) and are associated with increased functional deficits and impose a greater burden on the family (12, 27, 28). The most common neuropsychiatric symptoms are depression, irritability, apathy, anxiety, agitation, and sleep disturbances (26). Some neuropsychiatric symptoms, among other factors (29–31), have been observed to predict conversion from MCI to dementia (32–34). For instance, depression and apathy have been found to be more common in patients with MCI who were later diagnosed with AD (35). Addressing these symptoms may potentially reduce the likelihood of conversion to dementia (30).

Fortasyn Connect (Souvenaid®) is a specialized medical food that has demonstrated cognitive and behavioral benefits in patients with MCI and dementia, mainly due to AD (36–38). Significant decreases in decline have been observed across several measures including the Neuropsychological Test Battery (NTB) 5-item composite, NTB memory, Clinical Dementia Rating-Sum of Boxes (CDR-SB), memory brain atrophy (hippocampal, whole-brain and ventricular), Neuropsychiatric Inventory questionnaire (NPI-Q), Geriatric Depression Scale (GDS), and stabilization of everyday functioning as measured by Blessed Dementia Scale (BLS-D) and Rapid Disability Rating Scale 2 (RDRS2) (37, 38).

Developed to support the formation and function of neuronal membranes and synapses, Fortasyn Connect contains long-chain omega-3 fatty acids, uridine, choline, B vitamins, vitamin C, vitamin E, and selenium (37). Several studies have evaluated the effect of Fortasyn Connect in patients with MCI (36–40). However, the majority of these studies were either clinical trials (38, 41) or retrospective real-world studies (36, 37). Real-world prospective studies examining the effect of Fortasyn Connect on MCI are scarce.

Considering this context, we have designed a prospective real-life study, with the primary objective of evaluating changes in neuropsychiatric symptoms at 6 months after initiating Fortasyn Connect in patients recently diagnosed with MCI by a primary care physician. As secondary objectives, the following have been included: to determine changes in neuropsychiatric symptoms at 12 months after initiating Fortasyn Connect, to evaluate changes in HRQoL and cognition at 6 and 12 months, to describe the profile of patients recently diagnosed with MCI, and to assess the safety and persistence of Fortasyn Connect during the study period. As exploratory objectives, we also included the evaluation of changes in speech characteristics using a machine learning (ML) technique, a specific subset of artificial intelligence (AI), at 6 and 12 months, and the determination of the relationship between results obtained from traditional neuropsychological tests and those obtained using the ML technique at baseline, month 6, and month 12.

## 2 Methods and analysis

### 2.1 Design

**Figure 1:**
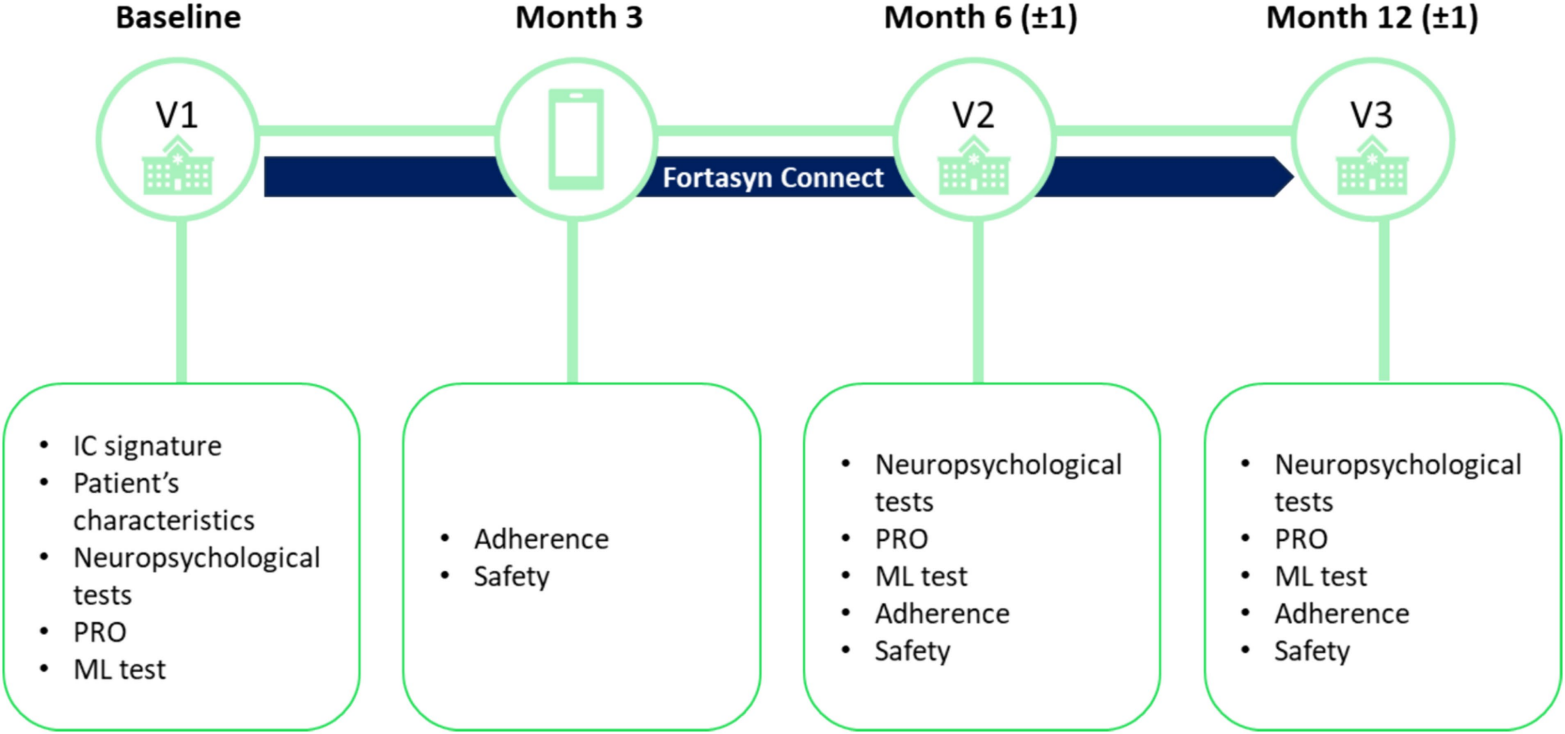
Study design. IC, informed consent; ML, machine learning; PRO, patient-reported outcomes.

This is an observational, prospective, multicenter study in primary care health centers across different regions in Spain (see Figure 1).

The study consists of three in-person visits, which will coincide with the patient’s visits to their primary care health center per clinical practice. The first study visit (baseline visit; V1) will be the visit in which the physician confirms that the patient meets the screening criteria, informed consent is signed, and the baseline assessment is performed. Visit 2 (V2) will be carried out at approximately 6 (±1) months and visit 3 (V3) at 12 (±1) months after the baseline visit. During these visits, the data necessary to meet the objectives of the study will be collected. In addition, at 3 months, a telephone call will be made to the patient to monitor adherence to treatment and safety.

The study has been approved by the Ethics Committees of Hospital Clínico San Carlos, Health Areas of Valladolid, Salamanca, Primary Care Management of Madrid, León and El Bierzo, Principality of Asturias and province of Córdoba.

### 2.2 Sample size estimation

A sample size of approximately 150 patients in 30 primary care centers has been estimated. The sample size calculation was based on determining a number of patients to achieve the primary objective of the study. The fulfilment of the secondary and exploratory objectives will be obtained from the sample size determined by the primary objective.

Using data from a study that assessed the effect of treatment on neuropsychiatric symptoms with the NPI-Q, it was established that the maximum variability of the change in the NPI score was around 17.63 points. Thus, with a sample of 150 patients, accepting an alpha risk of 5% and a beta of 20%, by means of a bilateral contrast, we can detect a change in the overall score on the NPI-Q of 4.15 points. A loss of information of 5%, including losses due to follow-up, of the data has been assumed.

### 2.3 Selection of patients

Patients eligible for inclusion in this study must meet all of the following criteria: (1) diagnosis within the last 6 months of MCI of undetermined etiology (subjective impression of a change in cognition [memory, attention, concentration, problem solving, or other] reported by patients, informants or clinicians; objective evidence of cognitive impairment in one or more cognitive domains; preservation of independence in functional abilities; not demented); (2) no prior pharmacological treatment or dietary supplement for MCI; (3) not having consumed Fortasyn Connect previously and being a candidate to receive it according to medical criteria, and as a shared decision between patient and the physician; (4) ability to understand and complete the informed consent (IC) and the questionnaires and tests of the study; (5) having signed the IC; (6) be accompanied by an informant, defined as a person with close and regular contact with the patient to observe and report changes in the patient’s behavior and other symptoms.

Exclusion criteria are: (1) participating in a clinical trial with experimental drug therapies or having participated in a clinical trial within the last 6 months; (2) presence of any severe psychiatric disorder, drug addiction, or alcoholism; (3) short life expectancy as judged by the physician or NECPAL+ criteria (42); (4) to have a caregiver due to functional deterioration and incapacity.

### 2.4 Outcomes

Study assessments per visit are summarized in Table 1. To ensure that the tests are conducted uniformly and the outcomes are collected consistently, a prior online training session will be held for the investigators involved in the study.

**Table 1:** Data collection schedule.

Data	Visit 1 (baseline, month 0)	Call (month 3 ± 1)	Visit 2 (month 6 ± 1)	Visit 3 (month 12 ± 1)
IC	x			
Selection criteria	x			
Demographic and clinical characteristics	x			
NPI-Q	x		x	x
Fototest	x		x	x
Clock drawing test	x		x	x
AcceXible test	x		x	x
EQ-5D	x		x	x
Adherence	x	x	x	x
Safety	x	x	x	x

IC, informed consent; NPI-Q, neuropsychiatric inventory questionnaire.

#### 2.4.1 Primary outcome measure

Changes in neuropsychiatric symptoms will be assessed by the NPI-Q. The primary outcome measure is mean change in NPI-Q score 6 months after starting Fortasyn Connect compared to baseline.

The NPI-Q was designed to evaluate neuropsychiatric symptoms in AD and other neurodegenerative disorders in routine clinical practice settings. The NPI-Q is completed by patients’ informants, and it covers 12 domains (delusions, hallucinations, agitation/aggression, depression/dysphoria, anxiety, elation/euphoria, apathy/indifference, disinhibition, irritability/lability, motor disturbance, sleep behavior, and appetite/eating) (43). Neuropsychiatric symptoms within each domain must be marked as present or absent. When a symptom is present, then it must be rated in terms of both frequency (1 = rarely, less than once per week; 2 = sometimes, about once per week; 3 = often, several times per week; and 4 = very often, once or more per day) and severity (1 = mild, 2 = moderate, 3 = severe), yielding a composite symptom domain score (frequency × severity). Caregiver distress is rated for each positive neuropsychiatric symptom domain on a scale ranging from 0 (not distressing at all) and 5 (extremely distressing).

#### 2.4.2 Secondary outcome measures

Changes in neuropsychiatric symptoms will be also assessed by the NPI-Q at month 12, as secondary outcome measure. Additionally, changes in cognition and HRQoL will be evaluated after 6 and 12 months of initiating Fortasyn Connect.

HRQoL will be evaluated using the paper-based version of the EQ-5D-5L, developed by the EuroQol Group (44). The questionnaire includes five dimensions reflecting generic HRQoL (mobility, self-care, activities of daily living, pain and discomfort, and anxiety and depression). Each dimension in the EQ-5D-5L has five response levels (no problems, slight, moderate, severe, and extreme problems). There are 3,125 possible health states defined by combining one level from each dimension. Health states are converted into a single index utility score with a scoring algorithm that ranges from-0.281 to 1, where values lower than 0 represent states considered to be worse than death (44). The tool also features a visual analog scale (EQ-VAS) that offers a unified assessment of self-perceived health. It is measured on a scale from 0 to 100 mm representing “the worst health you can imagine” and “the best health you can imagine,” respectively. The EQ-5D can be self-completed or administered by an interviewer. The 5 L version was created to improve the sensitivity of the 3 L version and reduce ceiling effects by increasing the number of severity levels (44). Numerous studies have used the EQ-5D-5L in patients with dementia and, therefore, the psychometric properties of the questionnaire in this population are known (45). The EQ-5D-5L usually takes less than 5 min to complete for the elderly (46).

Cognition will be assessed using the Fototest and the clock-drawing test, which are valid and widely adopted screening measures for MCI (47–50). Two cognitive assessment tests have been selected for measuring MCI because each test offers information into different cognitive domains and because cognitive scores should be interpreted collectively rather than relying on individual instruments. The clock-drawing test is a classic paper-based cognitive screening tool easy to administer in the clinical setting. It assesses various cognitive domains, including verbal comprehension, memory, spatial knowledge, abstract reasoning, planning, concentration, and visuoconstructive skills (51). The patient is required to draw a clock indicating the time as 11:10, and their performance is scored based on the depiction of the clock face (up to 2 points), the clock hands (up to 4 points), and the numbers (up to 4 points). There are several methods to administer the test, with the approach proposed by Thalmann in 2002 considered the most suitable for primary care due to its simplicity and brevity.

The Fototest evaluates language (naming), memory (free recall and cued recall), and executive function (naming fluency) in less than 3 min. It has been validated for its use in the follow-up of patients with cognitive impairment especially in contexts where evaluators may vary between visits, due to its high interobserver reliability, or with patients with limited educational backgrounds (50). A systematic review and meta-analysis of its diagnostic accuracy of the Fototest concluded that it can be considered suitable for detecting cognitive impairment in primary care settings (52).

The following demographic and clinical characteristics will be collected at baseline: age, sex, body mass index, years of schooling, and MMSE score. Concomitant treatment for MCI (both pharmacological and non-pharmacological), as well as comorbidities, will be collected at V1, V2 and V3. Adverse reactions and adherence to Fortasyn Connect will be assessed during in-person visits, as well as during the phone call at month 3. This phone call was included to provide closer patient follow-up and to ensure patient adherence to the treatment. The specific adverse reactions (diarrhea, nausea, abdominal discomfort, among others), their severity, onset, and resolution will be documented. If the adverse reaction is considered severe, the date when the reaction becomes severe, severity criteria, actions taken, and outcome will be recorded.

#### 2.4.3 Exploratory outcome measures

Changes in speech characteristics will be assessed after 6 and 12 months, using the AcceXible tool. AcceXible is a novel speech analysis algorithm designed to detect cognitive impairment, which has been validated in a Spanish population (53, 54). The relationship between the results obtained in traditional neuropsychological tests (Fototest and clock-drawing test) and those obtained with the ML technique (AcceXible) at baseline, month 6, and month 12 will be explored.

The AcceXible test consists of a computerized system with three independent yet interconnected modules: the data collection module (system interface for the user), the data processing module (detection algorithms), and the results output module. Communication between the different modules and data hosting is done in an encrypted and secure manner. For the construction of the detection ML model, acoustic and lexical semantic variables from three commonly used tests are considered: cookie theft, picture description, and semantic verbal fluency (*animals* category); an open-ended question that captures spontaneous speech is also included.

The audio recordings of the patient’s voice during the tests are collected, along with relevant information such as age and gender. These recordings are then sent to the AcceXible cloud. First, the audio is processed and transformed, and then passed to a trained initial model (Voice Model), which generates an estimation of the probability of cognitive impairment based on the patient’s voice. Simultaneously, the audio is run through an open-source Speech2Text API, which transcribes the audio from each test and returns the corresponding text. Next, the transcriptions of the tests are processed and passed through a trained model for each test. Each of these models generates a probability of impairment based on that particular test. Finally, these individual probabilities are combined with the patient’s general information available, and a final model generates the estimation of whether the patient has cognitive impairment.

The final model yields a result based on a classification system that assigns a patient the label of “healthy” or “impaired.” The AcceXible system also allows obtaining a score for each test, facilitating comparison of tests at different stages of patient monitoring. For the selection of the best model, a strategy called cross-validation is used. The metric used for validation and for selecting the appropriate classifier model is the area under the ROC curve (AUC). Once the best model is chosen, it is validated to calculate its generalization error. To do this, a strategy similar to cross-validation is used, but where K equals the number of patients, known as leave-one-out. Finally, with all predictions, the metrics that are calculated to provide an estimation of the model’s performance are accuracy, precision, recall and AUC.

### 2.5 Treatment

As this is an observational study designed to reflect real-world practice, Fortasyn Connect will be prescribed by the clinician, in agreement with the patient, and independent of the decision to offer the patient the option to participate in the study. Healthcare professionals may add or withdraw the treatment according to their clinical judgment.

Souvenaid® is a 125 mL once-a-day drink containing the specific nutrient combination Fortasyn Connect (Table 2). It will be taken as a supplement to the usual diet, for the duration prescribed by the primary care physician.

**Table 2:** Nutritional composition of Fortasyn Connect.

Nutrient	
Energy	125 kcal
Protein	3.8 g
Carbohydrate	16.5 g
Fat	4.9 g
EPA (eicosapentaenoic acid)	300 mg
DHA (docosahexaenoic acid)	1,200 mg
Phospholipids	106 mg
Choline	400 mg
UMP (uridine monophosphate)	625 mg
Vitamin E (α-TE tocopherol equivalents)	40 mg
Vitamin C	80 mg
Selenium	60 μg
Vitamin B12	3 μg
Vitamin B6	1 mg
Folic acid	400 μg

### 2.6 Data management

Once the IC has been signed and the patient’s eligibility confirmed, the investigator or designated personnel will start data collection. The investigator or designated personnel will be solely responsible for entering data into the electronic Case Report Form (eCRF), ensuring that the data recorded in the CRF is legible, accurate, and complete, and within the established timeframe. Data received through e-Clinical methodology will undergo timely workflows to comply with the 21 Code of Federal Regulations Part 11 Food and Drug Administration (FDA) standard, ensuring that electronically transmitted data are as valid as the original paper-based data. Furthermore, the eCRF will adhere to the following regulations: Good Clinical Practice (GCP) E6 R2; Good Manufacturing Practice (GMP) - EU: ANNEX 11 for Computerized Systems and ANNEX 15 for Qualification and Validation; GAMP Guide for Validation of Automated Systems in Pharmaceutical Manufacturing, Version: V5.0 - A Risk-Based Approach to Compliant GxP Computerized Systems; GAMP GCP Guide for Validation and Compliance of Computerized GCP Systems and Data; ISPE 2017; PIC/S: Good Practices for Computerized Systems in Regulated “GxP” Environment - September 2007; Regulation (EU) 2016/679 of the European Parliament and of the Council of 27 April 2016 on the Protection of Natural Persons with Regard to the Processing of Personal Data and on the Free Movement of Such Data (GDPR); the Taipei Declaration of the World Medical Association (WMA) in 2016.

An automated validation program will identify data discrepancies, enabling the modification or verification of data input by the investigator or designated personnel.

Patients included will be identified by a numerical code, ensuring that no personal data will be collected in the study database. The pseudonymization process will be carried out by the researchers participating in the study. The sponsor will, therefore, manage pseudonymized (encoded) data. This data will be stored at the center and will be kept in the investigator’s file.

An automatic validation program will check for data discrepancies, allowing modification or verification of the data entered by the investigator or designated personnel. Detected discrepancies, which require resolution, will be corrected by authorized personnel. Data clarification requests (‘queries’) describing the nature of the problem and requesting clarification for all other discrepancies and missing values may be created and forwarded to the research site. The designated research site staff should respond to the request for clarification and confirm or correct the data. Once these actions are completed and the database is declared complete and correct, the database will be closed, and the data will be available for analysis.

### 2.7 Data analysis

Statistical analysis will be conducted using the software *Statistical Package for the Social Sciences*, (SPSS) 22.0.

To analyze the primary outcome measure, the mean change in NPI-Q score at 6 months after initiating Fortasyn Connect will be calculated and compared to the baseline score. This comparison will be conducted using either the paired Student’s t-test or the Wilcoxon test, depending on the distribution of the data. Similarly, to assess the mean change from baseline in the NPI-Q score at month 12, the EQ-5D score at months 6 and 12, and the Fototest, clock-drawing test and AcceXible test at months 6 and 12, either the paired Student’s *t*-test or the Wilcoxon test will be used. To explore the correlation between the scores obtained on the Fototest and clock-drawing tests and those obtained on the AcceXible test at all time points, Pearson or Spearman correlation will be used, depending on the distribution of the data. Age will be included as a covariate in the analyses. The normality of the data will be tested using the Shapiro–Wilk test.

Patient characteristics and treatment safety and adherence will be summarized as mean and standard deviation (SD), median and interquartile range (IQR), or absolute and percentage frequency, as appropriate.

## 3 Discussion

As far as we know, this is the first prospective, multi-center study designed to assess the effectiveness of Fortasyn Connect in patients diagnosed with MCI in a real-world setting. Previous studies evaluating the effectiveness of Fortasyn Connect in the real-world have been single-center and either retrospective or prospective with a shorter follow-up period (up to 6 months) (36, 37, 55). The multicenter design of the present study facilitates a comprehensive representation of the MCI population in Spain, enhancing the generalizability of the study findings. Moreover, its prospective nature with four visits (one of them a telephone follow-up) enables the collection of more complete data from the study assessments. By conducting studies that monitor patients over an extended period, such as 12 months, researchers can assess the sustained effects of Fortasyn Connect on specific outcomes. This study will provide valuable insights into the long-term benefits of Fortasyn Connect in neuropsychiatric symptoms, cognition, and HRQoL complementing the results of prior observational studies and clinical trials.

Timely detection and effective management of MCI play a crucial role in maintaining patients’ functionality and HRQoL. However, evidence suggests that dementia detection rates remain notably low (15, 56). To address this, efforts to increase MCI detection in primary care need to be intensified (57). MCI is often misdiagnosed in primary care due to various barriers associated with evaluating cognitive performance. The Spanish Plan for Alzheimer’s and Dementias (2019–2023) aimed to improve early diagnosis of MCI by, among other measures, facilitating access to basic analytical and neuroimaging tests (such as CT scans) for primary care professionals (58). Despite these efforts, access to these tests remains limited, and diagnosis still relies on clinical interviews, patient’s medical history, and some cognitive assessments. Given the time restrictions in primary care consultations, there is a need for using brief and validated tests to objectively identify MCI within minutes (59). The tests included in the present study are widely used and easy to implement in MCI diagnosis and patient follow-up.

Although extensively used in clinical and research settings, the MMSE has limited accuracy for detecting MCI and is unsuitable for illiterate individuals (60–62). For these reasons, the Fototest and clock-drawing tests, as alternatives to the MMSE, will be used in the present study for assessing cognition at both diagnosis and follow-up. The Fototest has shown the same diagnostic performance as the Eurotest and Memory Alteration Test (M@T) but with a shorter administration time (Fototest: 2.8 min vs. Eurotest and M@T ~ 7 min) (63). In primary care, the tests administered must require minimal time, considering the consultation time is usually less than 10 min in Spain, a duration observed in various countries around the world (64). Assessments will be conducted at intervals of approximately 6 months in this study, which is the standard follow-up time of these patients in clinical practice. This time is adequate for mitigating practice effects that would artificially inflate scores in the tests. Three parallel versions of the Phototest (65) will be used to further reduce this practice effect.

Timely diagnosis in primary care not only allows for the identification of treatable causes of cognitive impairment, but also enables education for patients and families about the diagnosis, helps manage comorbidities and modify lifestyle, and enhances participation in research studies (18). While we are approaching a time when simple and accessible diagnostic tools, such as blood-based biomarkers for early detection of AD and other dementias, are anticipated to be available, we have not yet reached that point (66, 67). Currently, the use of biomarkers in clinical practice remains constrained, and they are mainly used in the clinical research context (68). As new biomarkers are expected to be progressively available in clinical practice, a European consensus on the use of biomarkers for the diagnosis of MCI is in preparation (69).

Digital biomarkers also hold potential in diagnosing MCI, offering objective and continuous monitoring of cognitive function through various technologies such as smartphone applications, wearable devices, and computer-based assessments. The AcceXible tool included in the present study uses ML techniques to identify individuals with MCI based on acoustic features of speech (70). These acoustic features have been associated with amyloid status (*β*-amyloid 1–42) assessed by cerebrospinal fluid in patients with MCI (71), suggesting their potential as a surrogate marker for underlying pathology.

The likelihood of progression from MCI to dementia is influenced by several risk factors, some of which are not modifiable (i.e., genetics) and others that are potentially modifiable, such as neuropsychiatric symptoms, diabetes, and low dietary folate (29–31). Specific neuropsychiatric symptoms in MCI may predict conversion to particular types of dementia. In a study of 2,137 patients with MCI, irritability and apathy predicted conversion to dementia, with irritability being the most discriminant symptom to identify non-AD converters (i.e., frontotemporal dementia, vascular dementia, Parkinson’s disease, and dementia with Lewy Bodies) (32). Neuropsychiatric symptoms are related to brain dysfunction and pathology in patients with MCI and dementia (72). Patients diagnosed with MCI who had Aβ deposition had an elevated risk of experiencing neuropsychiatric symptoms in contrast to MCI without Aβ burden.

Lower plasma nutrient levels have been found in AD patients (73). This suggested impaired systemic availability of several nutrients, potentially preceding protein and energy malnutrition, and highlighted the potential of nutrition in AD management (73). Interventions with multi-nutrient formulas, such as Fortasyn Connect, have demonstrated cognitive benefits among older adults, individuals with MCI, and those with dementia (36, 38, 41, 74–76). Fortasyn Connect has been shown to improve neuropsychiatric symptoms in MCI patients of unknown etiology as well as patients with dementia of various etiologies (36, 37), particularly in symptoms such as depression, anxiety, apathy, and irritability (37). However, despite these promising findings, some meta-analyses have reported no significant beneficial effects of Fortasyn Connect on cognition, functional ability, or overall clinical status (77). The present study will provide further evidence of the effect of Fortasyn Connect on neuropsychiatric symptoms and cognitive performance.

It is also important to highlight that although it is expected that most patients with MCI will have an AD-related etiology, patients will not undergo the extensive testing required to determine the etiology of MCI in the context of the study, as this is not conducted in the primary care setting. As a result, we will include all patients with MCI, regardless of the underlying cause. The nutrients included in Fortasyn Connect are not only relevant to AD but also have potential benefits in MCI due to vascular pathology. For instance, omega-3 fatty acids have anti-inflammatory properties and improve endothelial function, which could support cognitive function in patients with vascular MCI (78). Similarly, B vitamins are known to lower homocysteine levels, which are associated with a reduced risk of both vascular dementia and AD (79).

Interestingly, Fortasyn Connect has been shown to stabilize 18F-fluoro-deoxyglucose (18F-FDG-PET) scans in MCI patients, a measure of hypometabolism associated with early synaptic and neuronal dysfunction (80). This effect underscores the therapeutic potential of Fortasyn Connect in addressing synaptic dysfunction. Synaptic dysfunction and loss is one of the earliest pathological manifestations in patients with MCI and dementia (81–83), and shows a strong correlation with cognition (84). The timing of synaptic dysfunction and loss in relation to other pathological events of AD is not clear yet. Some authors have suggested that synaptic dysfunction may arise as a consequence of amyloidosis, tauopathy, inflammatory cascades, and additional pathological pathways (85), while others highlight the co-occurrence of these events and the potential contribution of synaptic dysfunction to amyloid-*β* accumulation in specific regions, such as the default mode network (86). Fortasyn Connect was designed to target the formation and function of synapses within the brain. Although the present study will not assess biomarkers of synaptic dysfunction, potential benefits observed in cognition and behavior might be hypothetically supported by these changes in synaptic function.

The treatment goal for MCI patients is not only to alleviate or at least delay cognitive and neuropsychiatric symptoms but also to improve HRQoL (87). Indeed, HRQoL has been ranked as the highest priority by patients with MCI and partner respondents in a survey, followed by self-efficacy, functional status, mood, and memory (88). Despite the importance given to HRQoL, the effectiveness of Fortasyn Connect on this measure has not been evaluated to date, as far as we know. The present study will provide, for the first time, evidence of the impact of this intervention on HRQoL for up to 12 months. We hypothesize that Fortasyn Connect will have positive in all the outcomes assessed in the study, including neuropsychiatric symptoms, cognition and HRQoL.

Up-to-date, disease-modifying therapies for AD approved by the FDA, lecanemab and donanemab, have not been approved by the European Medicines Agency (EMA) (89). In the meantime, the therapeutic options for MCI and early dementia remain the same as in the last years: acetylcholinesterase inhibitors (AChEI) and NMDA receptor antagonists. An advantage of Fortasyn Connect is that it can be used in combination with pharmacologic treatments. The use of concomitant treatments for MCI will, therefore, be collected during the study. The efficacy of Fortasyn Connect has been observed when used alone or combined with AChEI, antidepressants, or ginkgo biloba (37). Indeed, studies showed that the benefits of Fortasyn Connect were enhanced when used in combination with AChEI.

Additionally, the selection criteria of the present study do not prevent patients from receiving other non-pharmacological treatments that have proven effective, such as risk factor management, lifestyle changes (physical exercise, diet and sleep), and cognitive stimulation activities. After patients are initially diagnosed with MCI in primary care, they are referred to a specialist. However, there can be a considerable delay from the primary care visit to the specialist visit (90). During this waiting period, patients and their families face uncertainty and seek possible treatments. Offering Fortasyn Connect is appropriate as it has been shown to be effective and safe in this population (36–38). Importantly, Fortasyn Connect has demonstrated favorable effects on the CDR-SB, which reflects real-life performance and has been widely used as a primary outcome in anti-amyloid antibodies trials (91).

Recent data from Spanish clinical practice showed that after diagnosis of AD by a neurologist, 60% of patients were subsequently managed by primary care physicians (92). This finding underscores the critical role played by primary care providers in the continuum of care for patients with cognitive impairment. We advocate for a more consistent, reliable, and timely detection of MCI within primary care settings. Early identification of MCI allows practitioners to intervene proactively, potentially delaying functional decline and HRQoL impairment. To achieve this, fostering ongoing dialogue between primary care practitioners and dementia specialists is needed.

### 3.1 Study limitations

Our study has some limitations that must be acknowledged. First, the observational nature of the study does not allow for the drawing of definitive conclusions about causality. Since the study is not randomized and has no placebo control, we cannot exclude the influence of confounding variables or bias. An important potential confounding bias that might affect both the exposure (Fortasyn Connect) and the outcome (changes in neuropsychiatric symptoms, cognitive function, or HRQoL) is age. To address this potential bias, age will be included as a covariate in the analyses of the objectives. If further covariates (e.g., other pharmacologic or non-pharmacologic treatments) are observed in these patients, we will consider their inclusion as a covariate in the statistical analysis. Second, the frequency of measuring adherence (at 3, 6, and 12 months) might limit the capture of variability in adherence patterns over time and could underestimate or overestimate participants’ adherence. Nevertheless, high adherence was observed in prior studies, and therefore, similar levels of adherence could be expected here (55). Third, since biomarkers will not be evaluated as part of the study, distinguishing MCI due to AD from MCI due to other conditions will not be possible. However, Fortasyn Connect is effective and safe in MCI of unknown etiologies as well as in different types of dementia (37). Thus, benefits in MCI patients regardless of the underlying pathology are expected. No interim analyses are planned for the present study, and results are expected to be available by mid-2026.
